# Effectiveness of question prompt list interventions for patients with cancer: A systematic review and meta-analysis of randomized controlled trials

**DOI:** 10.1016/j.apjon.2025.100765

**Published:** 2025-07-25

**Authors:** Ruichen Han, Jiayi Liu, Jiarui Chen, Jinfeng Ding, Can Gu, Ni Gong, Jinnan Xiao

**Affiliations:** a Xiangya School of Nursing, Central South University, Changsha, China; b Xiangya Evidence-Based Healthcare Research Center, Central South University, Changsha, China; c JBI Xiangya Research Centre for Evidence-based Healthcare Innovation, Central South University, Changsha, China; d The Third Xiangya Hospital of Central South University, Changsha, China

**Keywords:** Cancer, Patient–physician communication, Question prompt list, Shared decision-making, Systematic review, Meta-analysis

## Abstract

**Objective:**

This study aimed to evaluate the effectiveness of question prompt list (QPL) interventions in patients with cancer and to synthesize the delivery characteristics of such interventions.

**Methods:**

A systematic search of five electronic databases was conducted for English-language randomized controlled trials published up to January 2025. Two independent reviewers performed study selection and data extraction. Eligible studies included cancer patients aged 16 years or older, with QPLs used in the intervention group to facilitate patient–physician communication. The Cochrane Risk of Bias 2.0 tool was used to assess study quality, and meta-analysis was conducted using RevMan 5.4 software.

**Results:**

A total of 302 records were identified, of which 14 studies (reported in 16 articles) met the inclusion criteria. Interventions were categorized into two groups: QPLs with instructions and QPLs without instructions. Pooled meta-analysis demonstrated that QPL interventions significantly enhanced patient engagement in shared decision-making, increased the number of questions asked, and improved the perceived helpfulness of the material. Compared to QPL alone, QPL with instructions further increased the number of patient-initiated questions and improved decision self-efficacy.

**Conclusions:**

QPL interventions with accompanying instructions showed superior effectiveness in promoting patient question-asking behavior and enhancing decision self-efficacy. This review underscores the potential of QPLs—particularly those with instructions—to improve patient–physician communication. Further research is warranted to refine these interventions and explore their role in reducing patient anxiety.

**Systematic review registration:**

PROSPERO: CRD42024594145.

## Introduction

Cancer constitutes a major contributor to the global disease burden.^1^ According to GLOBOCAN 2022, nearly 20 million new cancer cases, and 9.7 million deaths, are reported worldwide.^2^ The growing selection of novel therapies, tests, and rehabilitative care options requires increased patient involvement in decision-making.^3^ Shared decision-making is a patient-centered approach that can empower patients with cancer to make more informed treatment decisions.^4^ The process can allow patients to weigh risks and benefits,^5^ reduce decision conflict and regret, and improve communication.^6^

Effective patient-physician communication constitutes a foundational element of shared decision-making.^7^ However, patient-physician communication is frequently suboptimal.^8^ Patients with cancer might struggle to formulate questions or express concerns to physicians because they do not recall or fully understand the information given to them. Conversely, physicians often cannot fully comprehend patients’ needs, which can lead to a misunderstanding of the information desired by patients.^9^ Therefore, tailored tools are needed that encourage patients to ask questions in consultations while enabling physicians to deliver information that meets the unique needs of each patient.

A question prompt list (QPL) could potentially address the above-mentioned challenges. A QPL is a low-cost communication tool provided before consultation that consists of a series of structured questions.^10^ It helps patients consider the questions they want to ask their physicians, thereby helping physicians tailor consultations to address patients’ specific concerns.^11^ A QPL can ensure that patients to meet their desired information needs,^12^ engage patients to participate in shared decision-making,^13^ and ensure important treatment issues are not overlooked.^14^

In recent years, QPLs have been delivered through forms, drawings, interviews, and web videos showing QPL's efficacy in patient participation in decision-making.^15^^,^^16^ Research indicated that using a QPL in outpatient networks significantly helped patients with cancer to ask questions about their concerns, and promoted higher levels of self-efficacy with lower levels of distress.^17^ Additionally, patients who received QPL with instructions demonstrated that patients could ask more questions with healthcare providers and increase the satisfaction during the consultation.^18^ However, patients with QPL but no instructions showed QPL cannot improved patients to ask more questions during consultations.^19^ Therefore, the effectiveness of QPL may differ in types of QPL, making it difficult to provide clear guidance for clinical practice and studies based on current findings.

Previous reviews have evaluated the effectiveness of QPL. Several reviews indicated that QPL could enhance patients with cancer to ask questions,^20^ facilitate patient-physician communication quality,^21^^,^^22^ and improve the psychological and cognitive outcomes in these patients.^23^ However, these studies have summarized QPL interventions combined with patient-centered communication interventions, making it impossible to determine the role of QPL. For instance, interventions about physicians or patients communication training^24^, ^25^, ^26^ may influent the effect of QPL on communication. While one review^14^ summarized the effectiveness of QPL without such components, but due to the heterogeneity of studies, no meta-analysis was conducted. Moreover, these reviews did not compare the effectiveness of QPL only or with instructions. Furthermore, to explore the utility of current QPL interventions and to determine future research and clinical directions, it remains necessary to analyze patients' satisfaction with the information provided by QPL and whether QPL can induce long-term anxiety in patients.

Therefore, this study aimed to 1) assess the effectiveness of QPL on patient-physician communication in cancer care; 2) evaluate QPL's effectiveness in patients with cancer by assessing the effects of patient decision-making, and patients' psychological status; 3) how do different delivery methods (with/without instructions) impact QPL effectiveness, and 4) identify the characteristics of effective QPL interventions for patients with cancer.

## Methods

### Study design

This is a systematic review of randomized controlled trials. The review process is reported in accordance with the Preferred Reporting Items for Systematic Reviews and Meta-Analyses (PRISMA) guidelines.^27^ The study protocol was registered with the International Prospective Register of Systematic Review (PROSPERO) under registration number CRD42024594145.

### Literature search

A comprehensive literature search was conducted across five electronic databases: PubMed, CINAHL, PsycARTICLES, Scopus, and Web of Science. The search was restricted to English-language publications and randomized controlled trials. The coverage periods for each database were as follows: PubMed (from 1994), CINAHL (from 2012), PsycARTICLES (from 2011), Scopus (from 1985), and Web of Science (from 1993), with all searches concluding in January 2025. A combination of general keywords such as “question prompt” and “cancer” was used. Search terms were initially developed in PubMed and subsequently adapted for the other databases using a consistent search strategy. Detailed search results for each database are presented in Supplementary Table S1.

### Inclusion and exclusion criteria

PICO was compiled and used as a guide for inclusion criteria. 1) Population: Patients (more than 16 years of age) with a definite diagnosis of cancer by pathology or cytology, regardless of tumor type or stage. The inclusion criterion was set at 16 years of age or older, based on the international consensus that individuals in this age group possess legal capacity for autonomous medical decision-making.^28^ 2) Intervention: Interventions that use a QPL in the intervention group for patient-physician communication in consultation can be included, and interventions to enhance the use of QPL could also be included. 3) Comparison: The control group was placebo control, usual care, or standard care. 4) Outcomes: The outcomes included but were not limited to the primary outcome: effects of the QPL on communication; and the secondary outcomes: effects of the QPL on decision-making and psychological status. 5) Study design: randomized controlled trial.

The exclusion criteria were as follows: 1) Pediatric patients with cancer were excluded. For pediatric patients with cancer, interventions mainly involved both the child and their primary caregiver,^29^^,^^30^ who is likely to influence and dominate patients' communication with physicians, and their medical decision-making.^30^ And the use of QPL may differ as interventions sessions involved the caregivers. 2) Non-English-language articles. 3) Commentaries, protocols, meta-analyses, conceptual analyses, pilot studies, or feasibility studies. 4) Full texts (or missing major results) that could not be sought from the authors were excluded. 5) Interventions combined with other communication components such as communication skill training, communication materials were excluded. 6) Complex interventions not related to QPL such as education coaching or materials were excluded.

### Study selection

Duplicate records were identified and removed using EndNote 21. Subsequently, two independent reviewers (RCH and JYL) screened the titles, abstracts, and full texts of all retrieved articles according to the predefined inclusion and exclusion criteria. Any discrepancies during the screening process were resolved through discussion to reach consensus. If consensus could not be achieved, a senior expert (JNX) was consulted for adjudication.

### Study data extraction

The research team was responsible for data extraction and developed an extraction sheet. Two independent reviewers (RCH and JYL) in the team extracted and verified the accuracy of specific data from the studies. The extracted information included study characteristics (author names, publication years, study regions), patient characteristics (such as cancer type, cancer stage, and age), details of interventions (including framework, items, QPL contents, delivery sessions and duration, intervention facilitators, modality, and setting), as well as outcome measures, and results of the QPL interventions. When extracting data from the three articles reporting on the same study,^31^, ^32^, ^33^ we selected outcomes based on the source that provided the most detailed reporting and complete sample information. Specifically, for the number of questions asked by patients with cancer, we utilized data from Buizza et al.^32^ as it contained more detailed reporting of this outcome. Regarding anxiety outcomes, we referenced Buizza et al.^33^ because this publication utilized a complete sample size throughout their analysis. The information is provided in Supplementary Table S2. Disagreements during the literature screening process were resolved through discussion; if consensus could not be reached initially, senior experts (JNX, JRC, JFD, CG, and NG) were consulted for resolution.

### Appraisal of risk of bias

Critical appraisal was conducted independently by two reviewers (RCH and JYL) to assess the risk of bias, and quality of the studies by using the Critical Appraisal Checklist from Version 2 of the Cochrane Risk of Bias tool (RoB 2.0). The tool assessed five domains to identify potential biases: the randomization process, deviations from the intended intervention, missing outcome data, measurement of outcomes, and selection of reported outcomes. Based on these judgments, studies were classified as “low risk,” “some concerns,” or “high risk” regarding their overall risk of bias.

### Data synthesis and analysis

A narrative summarized was conducted to describe the characteristics of the included studies. A meta-analysis was conducted by following Cochrane's recommendation, using Review Manager 5.4 software. As outcome indicators were measured using different validated scales, standardized mean difference (SMD) with 95% confidence interval (CI) was calculated in this study. Reviewers contacted the original authors for missing or unsuitable data. If data remained unavailable, we used the reported data for estimation. The RevMan Calculator was employed to analyze data both within and between groups. We converted the median and the interquartile range according to the specified papers.^34^^,^^35^ The significance of *I*^2^ values determines quantitative heterogeneity. *I*^2^ values of 25%, 50%, and 75% corresponded to low, medium, and high variability, respectively. If *P* ​< ​0.10, *I*^2^ > 50%, a random effects model was chosen. If *P* ​> ​0.10, *I*^2^ < 50%, a fixed effects model was applied. Subgroup analyses were based on the different types of interventions and the time points of all measurements. In addition, we performed a sensitivity analysis to verify the robustness of the study's outcomes by excluding individual studies and recalculating results.

If a study had two intervention groups, we included the ones that met the study inclusion criteria in the meta-analysis. If multiple follow-up outcome points were measured in a study, we selected data as close to the time point of each study as possible in meta-analyses.

## Results

### Search results

**Fig. 1:**
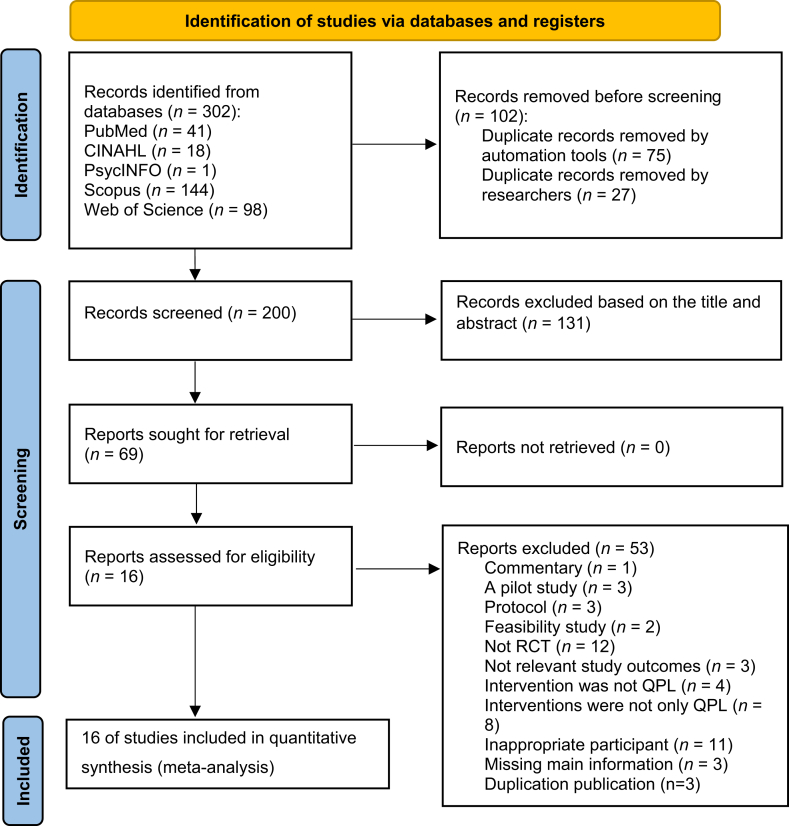
PRISMA flow diagram. PRISMA, Preferred Reporting Items for Systematic Reviews and Meta-Analyses.

In total, 302 articles were retrieved from the databases. Following the removal of 102 duplicates, 200 records were screened, and 69 studies were eligible for full-text review. After screening, 16 documents were identified as meeting the inclusion criteria, and the details of the reasons for the exclusion of studies at the final stage are provided in Supplementary Table S3. A total of 14 unique studies (reported across 16 articles) were included in the review, with three articles describing the same study but reporting different outcomes^31^, ^32^, ^33^ (Fig. 1).

### Study characteristics

Table 1 summarizes the characteristics of all studies. The 14 studies were conducted between 1999 and 2023. Five were conducted in Australia, three in the USA, and one each in Italy, French, German, Iran, Japan, and Chinese Taiwan. The studies encompassed 2678 patients diagnosed as having a variety of cancers, including breast cancer, esophageal cancer, and other multiple types of cancer, and 1409 (52.61%) of patients were female. Study sample sizes varied from 28 to 324, and the ages of patients ranged from 17 to 95 years old. The cancer stages ranged from 0 to IV.

**Table 1:** Summary of the characteristics, delivery details, and outcomes of studies.

Author and regions	Participants cancer type/cancer stage/age/sex	Sample size of participants	Outcomes and measurement Tools	Intervention facilitators	Modality	Setting
Negarandeh et al., 2023^36^ Iran	Breast cancer for follow-up treatment planning/unclear/50.04 ​± ​11.36 (39–65 years old)/all females	Baseline: I: *n ​=* ​25, C: *n ​=* ​25 Follow up: I: *n ​=* ​22, C: *n ​=* ​24	1. SDM-Q-9 (−); 2. CPS (−); 3. DSES (−)	A research assistant, researcher	Face-to-face contact, telephone or through the WhatsAPP social network/individual	In cancer center
Arthur et al., 2023^12^ America	Advanced cancer/unclear/58.59 ​± ​13.33 (44–72 years old)/79 females, 51 males	T0: Baseline: I: *n ​=* ​63, C: *n ​=* ​67 T1: Four weeks (± 7 days) after: An exploratory open-label format, *n ​=* ​47	T0 (after consultation immediately): 1. PAQ (−); 2. PSQ (−); 3. STAI (−); 4. Patient views of the information material and patient preference (+); 5. The total number and types of participant questions (−); 6. Speaking times (−); 7. Consultation duration (−) T1: /	Research staff, researchers at the outpatient palliative and supportive care clinic	1. Face-to-face contact: Patients openly received material 2. Face-to-face consultation Followed-up 4 weeks Individual	In cancer center
Tsai et al., 2022^13^ Taiwan	Breast cancer/0-IV/51.45 ​± ​9.64 (20–65 years old)/all females	T0: Baseline: I: *n ​=* ​120, C: *n ​=* ​120 T1 (1 week after): I: *n ​=* ​118, C: *n ​=* ​116 T2 (6 weeks after): I: *n ​=* ​115, C: *n ​=* ​115	T1: 1. DSES (+); 2. PEPPI (+); 3. PICS (+); 4. PrepDM (+); 5. mHCCQ (+); 6. CollaboRATE (−); 7. SURE (−); 8.DRS (−); 9. STAI-S (−); 10. Pain status scale (−); 11. Perceived health status scale (−) T2: DSES (+); 2. PEPPI (+); 3. PICS (−); 4. PrepDM (+); 5. mHCCQ (+); 6. SURE (−); 7.DRS (−); 8. STAI-S (−); 9. Pain status scale (−); 10. Perceived health status scale (−)	Two certified nurses and assistant who were trained, the details of training were unclear	1.Face-to-face consultation After 1 week and 6 weeks 2.Two assessments (face-to-face or telephone interview) to assessment outcomes Individual	In separate rooms
Buizza et al., 2021^32^ Italy	Breast cancer/I-III/56.22 ​± ​10.35 (43–68 years old)/all females	I: *n ​=* ​158; C: *n ​=* ​150	1. SWD (−); 2. SDM-Q-9 (−); 3. PEI (−); 4. PHQ-9 (+); 5. GHQ-12 (+); 6. CPS (−); 7. Number of question (+); 8. Consultation duration (−)	Research assistants, oncology nurse	Face-to-face consultation/can with caregivers	In the clinic waiting room or dedicated room (in each clinic)
Bouleuc et al., 2021^18^ French	Advanced cancer/unclear/59.66 ​± ​12.62 (28–92 years old)/109 females, 33 males	T0: Baseline: I: *n ​=* ​71, C: *n ​=* ​71 T1: After one month: I: *n ​=* ​45, C: *n ​=* ​51 T2: After two months: I: *n ​=* ​42, C: *n ​=* ​47	T1:/ T2: 1. McGill's scale (+); 2. HADS (−); 3. Brief-COPE (+); 4. PATSAT32 (+); 5. The total number of questions (+); 6. Topics of prognosis and EOL issues (+); 7. Consultation length (−)	PC team	Three times face-to-face consultations, interval of one month/individual	In three cancer centers
Zetzl et al., 2020^19^ German	Patients with cancer/unclear/64.9 ​± ​11.9 (25–95 years old)/129 females, 150 males	I: *n ​=* ​139, C: *n ​=* ​140	1. iE-Q (+); 2. Information needs: the question (−)	Unclear	Face-to-face consultation/individual	In waiting room
Buizza et al., 2020^33^ Italy	Breast cancer/I-III/56.31 ​± ​10.35 (46–67 years old)/all females	I: *n ​=* ​164, C: *n ​=* ​160	1. PDRQ-9 (+); 2. DDPRQ-10 (+); 3. STAI-X1/STAI-XI/Ra (−)	Research assistants, oncology nurse	Face-to-face consultation/can with caregivers	In the clinic waiting room or dedicated room (in each clinic)
Tattersall et al., 2017^37^ Australia	Patients with cancer in phase 3 cancer treatment trials/unclear/56.95 ​± ​13.78 (22–85 years old)/45 females, 43 males	T1: Baseline: I: *n ​=* ​45, C: *n ​=* ​43 T2: Within 3 weeks: I: *n ​=* ​39, C: *n ​=* ​28	T2: 1. STAI (−); 2. QuIC (−); 3. Physician satisfaction with the consultation (−)	Research nurse	Face-to-face consultation/individual	In cancer centers
Eggly et al., 2017^38^ America	Breast, colon, or lung cancer, self-identified as black, African American, or Afro-Caribbean/unclear/58.89 ​± ​10.35 (46–70 years old)/104 females, 9 males	T0: Pre-visit: I: QPL: *n ​=* ​42, QPL ​+ ​coach: *n ​=* ​36, C: *n ​=* ​44 T1: Post-interaction Intervention: QPL: *n ​=* ​40, QPL ​+ ​coach: *n ​=* ​34, C: *n ​=* ​40 T2: One week later: QPL: *n ​=* ​40, QPL ​+ ​coach: *n ​=* ​34, C: *n ​=* ​40	T1: 1. Patient perceptions of oncologist communication: a 4-point scale (+); 2. Consultation length (−); 3. Patient active participation in clinical interactions: Global ratings (−), frequency count (+), and oncologist-patient talk time ratio (−); 4. Patient role in treatment decision: a Nonlinear five-point scale (−); T2: 1. Patient perceptions of the intervention (−); 2. Patient trust in the oncologist (−)	Research staff	1. Face-to-face consultation After one week 2. A follow-up telephone interview assess their perceptions of the oncolorvention Individual	In outpatient clinics
Bottacini et al., 2017^31^ Italy	Early stage breast cancer/I-III/56.22 ​± ​10.35 (43–68 years old)/all females	I: *n ​=* ​158, C: *n ​=* ​150	1. Number of question (+); 2. Satisfaction with information: three following questions (+); 3. SWD (−); 4. STAI-X1/STAI-X1/R (−)	Research assistants, oncology nurse	Face-to-face consultation/can with caregivers	In the clinic waiting room or dedicated room (in each clinic)
Smets et al., 2012^39^ Australia	Esophageal cancer/unclear/65.34 ​± ​6.96 (52–82 years old)/7 females, 21 males	I: *n ​=* ​17, C: *n ​=* ​11	After two days: 1. Number of question (+); 2. Topics discussed (+); 3. Consultation length (−); 4. Satisfaction: a five-item questionnaire (−)	Unclear	1. Face-to-face consultation After two days 2. A telephone interview to assess the outcomes about the QPL and consultation Individual	In the center
Shirai et al., 2012^40^ Japan	Advanced cancer/II-IV/63.12 ​± ​10.56 (28–82 years old)/21 females, 42 males	I: *n ​=* ​32, C: *n ​=* ​31	1. Usefulness of the material(s): three questions (+); 2. Satisfaction with the consultation: Five items (−); 3. Number of question (−)	Researcher	Face-to-face consultation/individual	In the hospital
Clayton et al., 2007^41^ Australia	Advanced cancer/unclear/65.08 ​± ​13.30 (51–79 years old)/ 69 females, 105 males	T1: Within 24 hours after the consultation: I: *n ​=* ​92, C: *n ​=* ​82 T2: Primary analysis (three weeks after the consultation): I: *n ​=* ​90, C: *n ​=* ​80	T1: 1. Number of questions (+); 2. Number of items discussed by the QPL (+); 3. Patient satisfaction with the consultation: a 25 items adapted from Roter and Korsch et al. (−); 4. STAI (−); 5. Physician satisfaction: a 5-point Likert scale (−) T2: 1. STAI (−)	Research assistant	Face-to-face consultation/can with caregivers	Hospitals, inpatient PC units, and in homes, hostels, and nursing homes
Bruera et al., 2003^42^ America	Breast cancer/unclear/53.75 ​± ​10.75 (26–80 years old)/all females	I: *n ​=* ​30, C: *n ​=* ​30	1. Helpfulness of the information: a Numerical rating scale 0 to 10 (+); 2. Satisfaction with communication: the questions (−); 3. Number of questions (−)	Research nurses	Face-to-face consultation/individual	In cancer center
Brown et al., 2001^43^ Australia	Heterogeneous cancer/unclear/56.12 ​± ​16.25 (18–83 years old)/141 females, 177 males	T0: Baseline: I: Passive doctor ​+ ​QPS, *n ​=* ​79; I: Proactive doctor ​+ ​QPS, *n ​=* ​81 C: *n ​=* ​158 T1: After 7–10 days T2: Within 10 days	T1: 1. STAI (−); 2. Satisfaction with the consultation: a 25-item Likert scale adapted from Roter and Korsch et al. (−); 3. Number of questions (−); 4. Consultation duration (+) T2: ISQ (+)	Unclear	Face-to-face consultation/individual	In the hospital
Brown et al., 1999^44^ Australia	Patients with cancer/unclear/53 ​± ​15 (17–77 years old)/31 females, 29 males	T0: Baseline: I: QPS group, *n ​=* ​20 Intervention: QPS ​+ ​coaching, *n ​=* ​20, C: *n ​=* ​20 T1: After 7–10 days	T0 (after consultation immediately): 1. STAI (−); 2. Number of questions (−) T1: 1. Satisfaction: a 25-item Likert scale adapted from Roter and Korsch et al. (−); 2. MAC (−)	Researcher, research psychologist	Face-to-face contact/unclear	In the hospital

(−): the result was no statistically significant; (+): the result was statistically significant.

I, intervention group; C, control group; SDM-Q-9, 9-item Shared Decision-Making Questionnaire; CPS, Control Preferences Scale; DSES, Decision Self-efficacy Scale; PAQ, Patient Assessment Questionnaire; PSQ, Patient Satisfaction Questionnaire; STAI, Spielberger State-Trait Anxiety Inventory; GIS, General Information Sheet; PEPPI, Perceived Efficacy in Patient–Physician Interactions scale; PICS, Perceived Involvement in Care Scale; PrepDM, Preparation for Decision-Making scale; mHCCQ, modified Health Care Climate Questionnaire; CollaboRATE, CollaboRATE scale; SURE, Sure of myself, Understand information, Risk-benefit ratio, Encouragement test; DRS, Decision Regret Scale; STAI-S, State-Trait Anxiety Inventory-S; SWD, Satisfaction with decisions made during the consultation; PEI, Patient Enablement Instrument; PHQ-9, Patient Health Questionnaire Depression scale; GHQ-12, General Health Questionnaire; McGill, McGill Quality of Life questionnaire; HADS, Hospital Anxiety and Depression Scale; PATSAT32, patient satisfaction with cancer care questionnaire; EOL, end-of-life; iE-Q, Interactional empowerment questionnaire; PDRQ-9, Patient-Doctor Relationship Questionnaire; DDPRQ-10, Difficult Doctor-Patient Relationship; STAI-X1, Spielberger State-Trait Anxiety Inventory-X1; STAI-X1/R, a modified version of theSTAI-X1; QuIC, Quality of Informed Consent questionnaire; QPL, question prompt list; DT, Distress Thermometer; QPS, question prompt sheet; ISQ, Cassileth Information Styles questionnaire; MAC, Mental Adjustment to Cancer Scale.

### Risk of bias and quality assessment

**Fig. 2:**
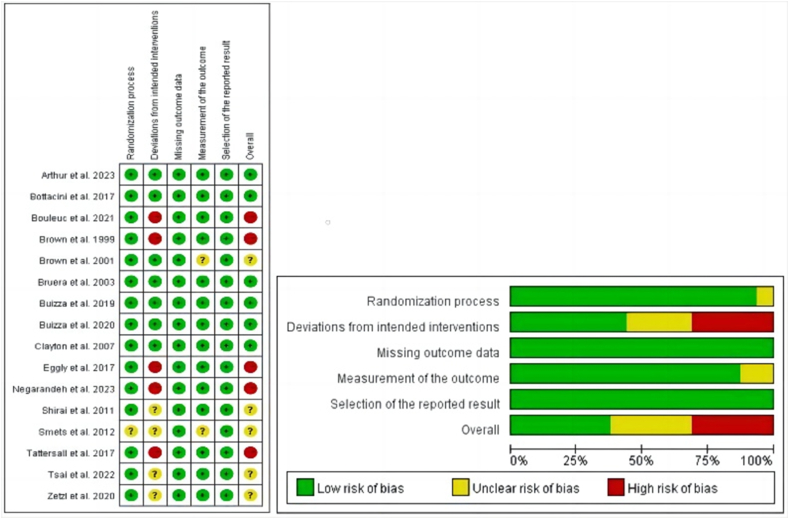
Summary of risk bias of the included studies.

Fig. 2 summarizes of the risk of bias across various domains in the included 14 studies. Of the 16 articles, six were rated as having a low risk of bias, five were rated as having an unclear risk of bias and five were rated as having a high risk of bias. Five studies exhibited a high risk of bias regarding deviation from established interventions, due to the lack of blinding of subjects and intervention providers, causing data to be unbalanced and missing.

### Intervention characteristics

Table 1, Table 2 summarizes the intervention characteristics of the included studies.

**Table 2:** Summary of intervention characteristics of the included studies.

Interventions	Author	Theory	Methods for developing the QPL	Items and contents of QPL	Delivery session(s) and duration
**QPL only**	Negarandeh et al., 2023^36^	Self-efficacy theory	Based on existing evidence, expertise, patients	The QPL: Following surgery for **breast cancer** A total of 14 questions, three domains: 1. Information about the malignancy; 2. Treatment options; 3. Choices for follow-up after treatment.	Session 1: Patients received QPL Session 2: Patients asked to mark questions in the QPL Session 3: Patients received a follow-up phone call to remind them to use the QPL The duration is unclear
	Buizza et al. 2021^32^	Unclear	The QPS of study based on English QPS developed by the Australian group	The QPS: A total of 50 questions. 1. topics; 2. Diagnosis; 3. Treatment; 4. Contribution of the patient and lifestyle; 5. Prognosis; 6. Other issues	Session 1: Patients received the QPS The duration is unclear
	Zetzl et al., 2020^19^	Unclear	Combined the QPLs with emotional support of the patients, based on the medical team	The QPL: Contains physical complaints, need for information on support and palliative care. 1. QPL includes implicit question prompts (iQP) to express emotions; 2. QPL includes explicit question prompts (eQP) to improve the explicit expression of emotions.	Session 1: Patients received a written QPL The duration is unclear
	Buizza et al., 2020^33^	Unclear	The QPS of study based on English QPS developed by the Australian group	The QPS: A total of 50 questions. 1. topics; 2. Diagnosis; 3. Treatment; 4. Contribution of the patient and lifestyle; 5. Prognosis; 6. Other issues	Session 1: Patients received the QPS The duration is unclear
	Bottacini et al., 2017^31^	Unclear	The QPS of study based on English QPS developed by the Australian group	The QPS: A total of 50 questions. 1. topics; 2. Diagnosis; 3. Treatment; 4. Contribution of the patient and lifestyle; 5. Prognosis; 6. Other issues	Session 1: Patients received the QPS The duration is unclear
	Tattersall et al., 2017^37^	Unclear	The transcripts were analyzed using rigorous qualitative methodology, based on patients with cancer, and expertise	The QPL: A total of 51 questions, 10 headings. Headings: 1. Understanding my choices; 2. Finding out about this trial; 3. Understanding the trial's purpose and background; 4. Understanding the possible benefits; 5. Understanding the possible risks; 6. The differences between going on the trial & having standard treatment; 7. Understanding how the trial is being carried out; 8. Understanding “randomistation”; 9. Understanding possible conflicts of interest; 10. Understanding my right to join or to join trial	Session 1: Patients received the QPL and had few minutes to review it The duration is unclear
	Smets et al., 2012^39^	Unclear	A validated question prompt sheet (McJannett et al., 2003) was independently translated into Dutch by two translators	The QPL: **For surgical patients** A total of 38 questions, covering the domains of: 1. Diagnosis; 2. Tests; 3. Prognosis; 4. Treatment options; 5. Multidisciplinary team; 6. Surgery; 7. Effects of surgery; 8. Quality of life; 9. Support information	Session 1: Patients received the QPL Session 2: 15 minutes to read and consider the questions about the QPL More than 15 minutes
	Bruera et al., 2003^42^	Unclear	Developed by previous studies and other suggestions	The QS: **for breast cancer** The prompt sheet contained breast cancer diagnosis, treatment, prognosis-related questions, and space for new questions. A total of 22 questions.	Session 1: Patients received the PS The duration is unclear
	Brown et al., 2001^43^	Unclear	In conjunction with the reader	The QPS: A total of 17 questions, the categories include: Diagnosis, tests, treatment, prognosis, psychological issues, support services available	Session 1: Patients received the QPS 15–20 minutes before consultation More than 15 minutes
**QPL with instructions**	**Arthur** et al., 2023^12^	Unclear	A Delphi process	The QPS: A total of 25 items, five domains, it contains general patient information about **palliative care and other related** information: Domains: 1. The palliative care service; 2. Symptoms, treatment and lifestyle; 3. Types of support; 4. End of life issues; 5. For caregivers	Session 1: Patients received QPS 30 minutes prior the consultation Session 2: Patients encouraged to read the information material before consultation Session3: Physicians asked to endorse the use of the information material by asking patients if they have any questions, explain the importance of asking questions More than 30 minutes
	Tsai et al., 2022^13^	The theoretical framework proposed by Taylor et al. (Taylor et al., 2017)	Based on the question list, expertise and patients	Patients with a six-page printed QPL booklet on **breast cancer**. The QPL, contains 15 topics: 1. Diagnostic exams; 2. Treatment strategies; 3. Surgery options; 4. Breast reconstruction; 5. radiation therapy; 6. Chemotherapy; 7. Hormone treatment; 8. Targeted therapy; 9. Traditional Chinese medicine treatments; 10. rehabilitation; 11. Nutrition; 12. Follow-up issues; 13. Social resources; 14. Pregnancy; 15. End-of-life palliative care	Session 1: Patients received QPL booklet Session 2: Patients selected three topics from booklet and research assistant explained it thoroughly Session 3: Surgeon endorsement, but details were unclear in the article The duration is unclear
	Bouleuc et al., 2021^18^	Unclear	Adapted an American or Australian QPL to the French, based on expertise and advanced patients	The QPL: **In the PC setting** The French version of the QPL is a 16-page A5 booklet, a total of 112 questions structured into 11 sections exploring the following 10 domains. Domains: 1. The supportive care and PC team; 2. Symptoms; 3. Anticancer treatments; 4. Symptom management; 5. Lifestyle and QOL; 6. Expectations and prognosis; 7. Finding support; 8. Health care quality; 9. Carer support; 10. EOL issues; and other questions	Session 1: Patients received the QPL booklet after the first consultation Within one month Session 2: Patients with the instruction to read it at home and to select items they would like to ask Session 3: Physicians had to endorse the use of the QPL with a standardized script to invite patients to ask questions in the QPL The duration is unclear
	Shirai et al., 2011^40^	Unclear	Based on previous QPS studies, patients and oncologists	The QPS : A 10-page A4 sheet. A total of 53 questions, 10 topics and a space for new questions. Topics: 1. Diagnosis; 2. Condition of a disease; 3. Symptom; 4. Test; 5. Treatment; 6. Life; 7. Family; 8. Psychological issues; 9. Prognosis; 10. Other issues	Session 1: Patients received the QPS Session 2: Patients were instructed to read the materials before the consultation The duration is unclear
	Eggly et al., 2017^38^	Unclear	Developed by black patients and family members, community members, and oncologists (Eggly et al., 2013)	The QPL: **to patients with low levels of education and health literacy.** A total of 43 questions, related to topics about: 1. Diagnosis; 2. Treatment; 3. Chemotherapy; 4. Side effects; 5. Daily life during treatment; 6. Treatment plan and schedule; 7. Help with costs; 8. Help with coping	Intervention 1: Session 1: Patients received the QPL Session 2: With a brief explanation and encouragement to read it Within two weeks
	Clayton et al., 2007^41^	Unclear	Developed by groups and individual interviews with patients, PC health professionals (Clayton et al., 2003)	The QPL: **targeting the end-of-life setting** The French version of the QPL is a 16-page A5 booklet, a total of 112 questions structured into 9 topics. 1. the palliative care service and team; 2. Physical symptoms; 3. Treatment; 4. Lifestyle and quality of life; 5. My illness and what to expect in the future; 6. Support; 7. If you are concerned about your professional care; 8. Caregiver; 9. End-of-life issues	Session 1: Patients received the QPL about 20 minutes before consultation Session 2: PC physician asked to actively endorse And refer to the QPL to encourage patients to ask questions and emphasize the importance of asking questions from the QPL More than 20 minutes
	Brown et al., 1999^44^	Unclear	Developed by experts and were grouped according to using a method of categorization described by Ley et al. (1973)	The QPS: A total of 17 questions, the categories include: Diagnosis, tests, treatment, prognosis, psychosocial issues, support services available	Intervention 1: Session 1: Patients received the QPS Session 2: Doctor endorsed the prompt sheet and went through each category eliciting and answering questions according to a standard protocol The duration is unclear

QPL, Question Prompt List; iQP, implicit question prompts; eQP, explicit question prompts; PC, palliative care; QPS, Question Prompt Sheet; GI, general information; QOL, Quality of life; EOL, end of life.

#### Theoretical bases of the interventions

Two theoretical models were referenced: self-efficacy theory, and the theoretical framework proposed by Taylor et al.,^45^ which emphasizes relational autonomy and self-determination. Both theories emphasize the importance of individual beliefs, autonomy, and multifaceted influences in communication. Self-efficacy theory aimed to enhance patients perceived capacity to participate in their treatment planning and decision-making process by providing them with QPL. While QPL interventions based on the theoretical framework proposed by Taylor et al.^45^ focused on providing QPL to patients and physicians before their consultations. It can enable patients to prepare questions, as well as help physicians understand patients' preferences. However, the link between theories and QPL were not clearly described.

#### Classification and content of interventions

The interventions can be classified into the following two categories based on differences in the intervention methods and materials: (1) QPL only and (2) QPL with instructions. QPL only include providing the QPL directly to the patients to read the QPL and ask questions based on the items themselves, with no explanation by intervention facilitators. QPL with instructions are QPL combined with instructions that help patients and healthcare providers understand about the QPL, improve both utilization of the QPL. QPL with instructions contain components for both patients and healthcare providers. Components for patients include explaining the items about the QPL prior to the consultation. Interventions for healthcare providers include getting them to endorse or use the QPL before the consultation with patients. This will enable them to emphasize the importance of the QPL to patients and to interpret patient information accurately.

QPL contents are categorized into three types based on the target population's characteristics: (1) palliative care patients with cancer; (2) patients with a specific cancer, such as breast; and (3) surgery patients with cancer. Common content including treatment, diagnosis, prognosis, and symptoms was identified in these QPLs. However, the focus varies depending on the target population. Those intended for palliative care patients with cancer focus on issues surrounding end-of-life care and hospice services. The QPL for patients with a specific cancer is tailored and addresses knowledge and symptom management for cancers. Similarly, the QPL for surgery patients with cancer concentrates on surgical procedures.

#### Intervention session and duration

The intervention sessions ranged from one to three sessions, and the time scale of sessions ranged from a few minutes to two months. The initial session was designed for patients to receive the intervention materials, and subsequent sessions were aimed at strengthening the QPL or encouraging patients to use the QPL during the consultation.

#### Intervention delivery modality

All 14 studies reported the intervention delivery modality. Most of the interventions were conducted face-to-face in hospitals, clinics, or cancer centers. Only one study specifically noted the option of utilizing face-to-face contacts, telephone, or WhatsApp social network tools, while another one study conducted interventions on hospitals, inpatient PC units, homes, hostels, or nursing homes, but the number of patients within each setting was unspecified. The majority of patients communicated with healthcare providers individually, two studies indicated that caregivers can participate in consultations with patients.

#### Intervention facilitators

Eleven studies described the facilitators of the interventions. Five studies were conducted by individual facilitators, including research assistants, researchers, and nurses. Five studies involved multiple facilitators, including social workers, coaches, and psychologists, in addition to researchers and nurses. Only one study reported that interventions were delivered by teams, which focused on palliative care. One study reported that intervention facilitators received training in groups, but the training specifics for the study were unclear.

### QPL effectiveness

The measurements were evaluated at three time points: immediately, about one week, and more than one week after interventions. The effectiveness of the studies was measured using 29 scales or questionnaires **(**Supplementary Table S4**)** in all, which can be categorized into the following three domains: effects of the QPL on communication, effects of the QPL on decision-making, and effects of the QPL on psychological status. To assess the effectiveness of different types of QPL, we performed subgroup analysis in most outcomes, otherwise the number of studies was less than two.

#### Effects of the QPL on communication

The effects of a QPL on communication can be categorized into three domains, namely the number of questions asked, consultation length, and patient-physician communication.

**Fig. 3:**
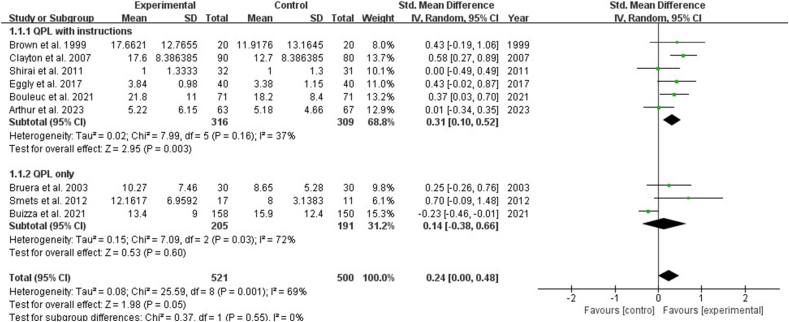
Impact of QPL on number of questions asked by patients with cancer. QPL, question prompt list.

**Fig. 4:**
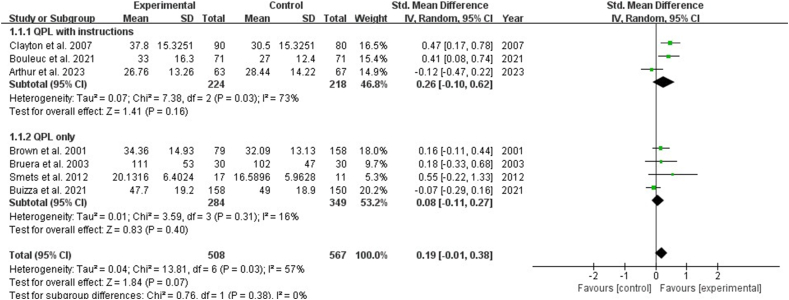
Impact of QPL on the consultation length in patients with cancer. QPL, question prompt list.

**Fig. 5:**
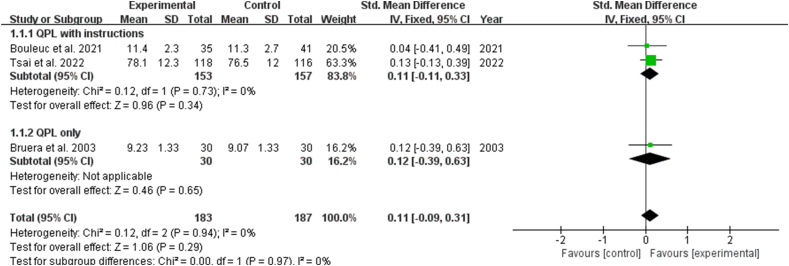
Impact of QPL on patient-physician communication. QPL, question prompt list.

Nine studies reported the impact of interventions on the number of questions by patients with cancer. The result showed that the QPL increased the number of questions asked by patients [SMD: 0.24, 95% CI (0.00, 0.48), *P* ​= ​0.05]. Subgroup analysis showed that QPL with instructions showed a significant increase in the number of questions asked by patients [SMD: 0.31, 95% CI (0.10, 0.52), *P* ​= ​0.003], while QPL only indicated no significance in the number of questions asked by patients (*P* ​> ​0.05) (Fig. 3). Seven studies reported the impact of the QPL on the consultation length for patients with cancer and revealed no statistically significant difference (*P* ​> ​0.05) (Fig. 4). Three studies described the patient's perceptions of patient-physician communication quality and revealed no statistically significant difference (*P* ​> ​0.05). (Fig. 5).

In sensitivity analysis, the exclusion of the study by six studies^18^^,^^38^^,^^39^^,^^41^^,^^42^^,^^44^ led to contradictory results in the number of questions asked. And excluding two studies^12^^,^^32^ caused contradictory studies in consultation length, indicates that the robustness and reliability of the meta-analysis results are suboptimal. For patient-physician communication, the results of the meta-analysis remained generally consistent when the individual studies were excluded one by one, suggesting the robustness and reliability of the meta-analysis results **(**Supplementary Table S5).

#### Effects of the QPL on decision-making

The effects of a QPL on decision-making can be categorized into four domains: shared decision-making, decision self-efficacy, decision satisfaction, and helpfulness of the material.

**Fig. 6:**
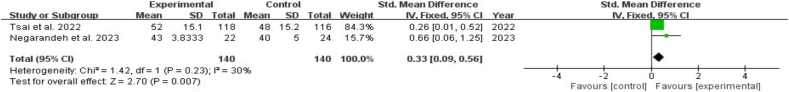
Impact of QPL on shared decision-making in patients with cancer. QPL, question prompt list.

**Fig. 7:**
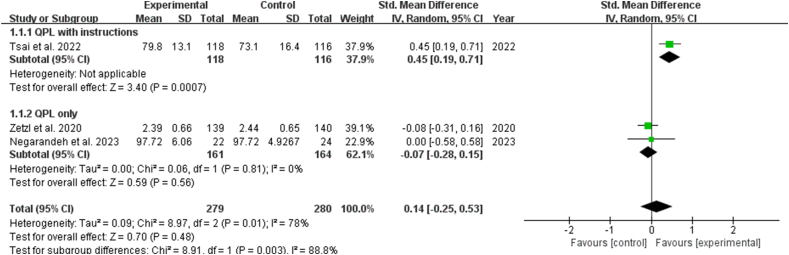
Impact of QPL on decision self-efficacy in patients with cancer. QPL, question prompt list.

**Fig. 8:**
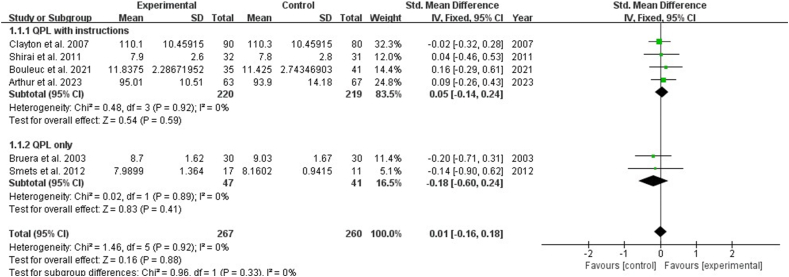
Impact of QPL on satisfaction with the consultation in patients with cancer. QPL, question prompt list.

**Fig. 9:**
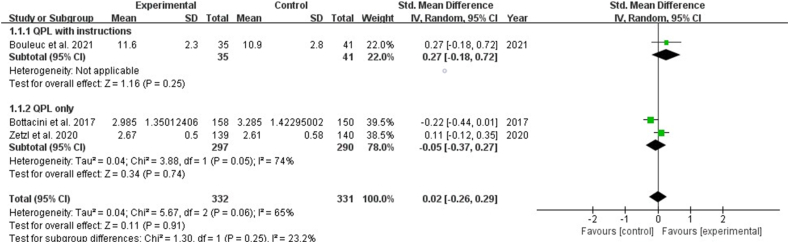
Impact of QPL on satisfaction with the information in patients with cancer. QPL, question prompt list.

**Fig. 10:**
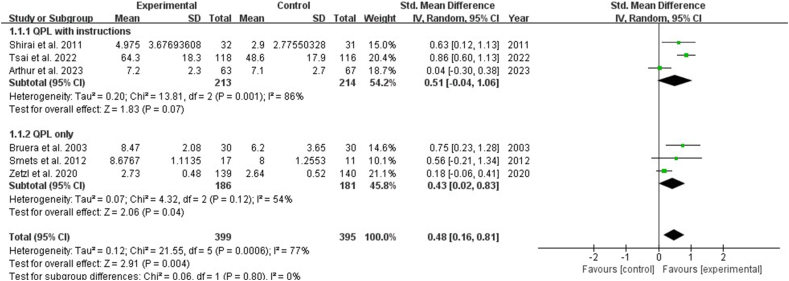
Impact of QPL on helpfulness of material in patients with cancer. QPL, question prompt list.

Two studies highlighted the effects of shared decision-making in clinical encounters, indicating that the QPL significantly increased it [SMD: 0.33, 95% CI (0.09, 0.56), *P* ​= ​0.007] (Fig. 6). Three studies reported on decision self-efficacy, indicating the effectiveness of the QPL for patients. The results revealed no statistically significant difference (*P* ​> ​0.05). Subgroup analysis showed that QPL with instructions showed significant effects in improving patients' decision self-efficacy [SMD: 0.45, 95% CI (0.19, 0.71), *P* ​= ​0.0007], while QPL only had no statistically significant effect on decision self-efficacy (*P* ​> ​0.5) (Fig. 7). Eight studies reported the effects of interventions on satisfaction and indicated no significant effect on satisfaction with consultation, or information (*P* ​> ​0.05) (Fig. 8, Fig. 9). Six studies discussed the helpfulness of the interventions. The results indicated a significant effect on the perceived helpfulness of the material [SMD: 0.48, 95% CI (0.16, 0.81), *P* ​= ​0.004]. Subgroup analysis showed that QPL with instructions demonstrated no statistically significant difference in the helpfulness of material (*P* ​> ​0.05), while QPL only improved the helpfulness of material [SMD: 0.43, 95% CI (0.02, 0.83), *P* ​= ​0.04] (Fig. 10).

In effects of the QPL on decision-making, the results of the meta-analysis remained generally consistent when the individual studies were excluded one by one, suggesting the robustness and reliability of the meta-analyses results.

#### Effects of the QPL on psychological status

**Fig. 11:**
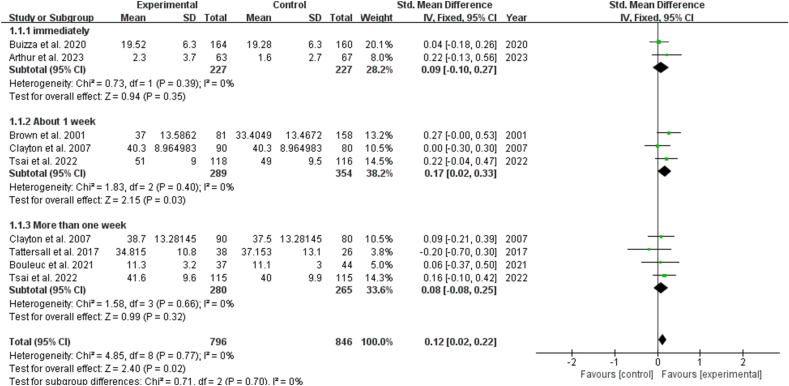
Impact of QPL on anxiety in patients with cancer. QPL, question prompt list.

The effects of a QPL on psychological status only included anxiety. According to previous study,^12^ anxiety was influenced by the time point of measurement, and we performed subgroup analyses based on multiple follow-up outcomes points. The results indicated a significant effect on the anxiety [SMD: 0.12, 95% CI (0.02, 0.22), *P* ​= ​0.02]. Subgroup analysis showed that QPL interventions increased anxiety after one week of using [SMD: 0.17, 95% CI (0.02, 0.33), *P* ​= ​0.03] (Fig. 11). Oher time points demonstrated no statistically significant difference (*P* ​> ​0.05).

Sensitivity analysis showed that excluding the studies by two studies,^13^^,^^43^ inverted the pooled results.

## Discussion

### Main findings

This systematic review and meta-analysis evaluated 14 studies assessing the effects of a QPL on communication between patients with cancer and healthcare providers, decision-making, and psychological outcomes for patients. Two categories of interventions, namely QPL with instructions and QPL only, were identified. Overall, QPL interventions improved patient engagement in shared decision-making, increased the number of questions asked by patients, and enhanced the helpfulness of material. Compared with QPL only, QPL with instructions showed superior performance in helping patients ask healthcare providers questions, boosting self-efficacy. QPL only made patients feel helpful of the material. However, QPL have been demonstrated to induce anxiety in patients for a period of time.

QPL increased the number of questions patients asked without increasing consultation time. The outcomes were consistent with the conclusion of previous study.^22^ QPL encompasses the information for patients and healthcare providers to enhance patient utilization of the QPL.^46^ In particular, some contents in QPL provide disease-related information, which could help patients gain insight into their disease and prepare adequately. It means QPL supports patients by providing treatment information and meeting patients' needs for relevance and autonomy in order to encourage patients to ask individualized and important questions.^19^ The process improves treatment self-efficacy and enhances patients' perceptions of their capability to participate in decision-making.^13^ However, the number of questions asked by patients was also related to how careful they were in reading the QPL. Our findings revealed that QPL only did not significantly impact the number of questions raised, which is consistent with the results of McDarby's study.^47^ QPL with instructions offer thorough explanations about the QPL and then allow patients to select or mark the questions before the consultation, which may help patients carefully read the QPL, summarize their concerns, and select the questions about themselves. This indicates that be given the QPL to patients, it is necessary to provide patients with clear instructions to help them understand its purpose, basic contents, and specific details, thereby enabling them to formulate more effective questions.

QPL did not significantly improve patient-physician communication, satisfaction with consultation, or satisfaction with information, which is contrary to expectations. This result suggests that the effectiveness of QPL requires further exploration. The overall quality of patient-physician communication is not only influenced by the content of QPL but also depends on the physician's communication skills, the mutual understanding of the QPL, and the ability to address patients' psychological needs. Simply providing QPL may undermine the significance of its components.^24^^,^^25^ Epstein's study found that physicians and patients received QPL-related training, coaching, and psychological counseling, quality of patient-physician communication increased.^24^ In clinical practice, simply providing the QPL may not be sufficient to meet the needs of both parties. While QPL primarily provides the necessary information, its efficacy depends on healthcare providers and patients to utilize this information effectively. However, QPL with instructions for healthcare providers most included getting them to endorse or use the QPL, healthcare providers may need to spend more time understanding QPL, which does not ensure effective communication with patients, it is crucial for every physician to have a one-time training session about QPL, which should cover communication skills, QPL usage, and its significance. Despite the clinical emphasis on the impact of a QPL on patients, few studies have assessed their utility to healthcare providers and their satisfaction with them. In the studies, we included tests mostly focused on physicians' perceptions of satisfaction, the helpfulness of the material, and physician-patient communication. Only one study^41^ showed that physicians thought the materials could help them with consultations, but others^12^^,^^37^ showed no change in physicians' attitudes between the intervention and control groups. Future research could concentrate on the factors influencing patient-physician communication and patient satisfaction, and assess the usefulness of QPL.

Our review found that QPL only made patients ‘feel’ the helpfulness of materials but QPL with instructions showed no statistically significant difference from controls. For patients with cancer, the majority of QPL were in paper format and situated in offline hospitals or cancer centers, and interventions might include multiple time points, which require patients to process and receive extensive information, potentially imposing a certain burden on patients. Simultaneously, QPL with instructions are mostly designed to introduce patients to QPL within a limited timeframe. Although these interventions provide detailed presentations, patients may struggle to read the full content and process the information thoroughly within a limited time. Consequently, during clinical consultations, patients' questions and healthcare providers' responses may deviate from anticipated outcomes, as prior explanations may have heightened patients' psychological expectations.^14^ Therefore, to improve the effectiveness and promote the use of QPL in clinical oncology, there is a need to reliably evaluate the QPL instruction that is better tailored to patient needs and more readily accepted. For instance, web-based interventions^15^ that allow patients to personalize the timing and duration of the intervention in a home environment may address QPL delivery issues. Furthermore, establishing evidence-based guidelines for optimal QPL delivery timing is essential to ensure patients have sufficient time to review the QPL and reflect on the questions it raises. Some patients report that the limited time available prevents them from fully understanding the QPL or using it to ask relevant questions.^48^ Therefore, researchers should assess the optimal timing for delivering the QPL to patients.

QPL may have inadvertently increased patient anxiety at one week, while QPL does not induce persistent anxiety. Anxiety may be caused by the content of the QPL, which heightens patients' awareness of their condition. Items of QPL focused on end-of-life, diagnosis, treatment, and prognosis. Information overload could contribute to cognitive burden as patients asked questions by QPL, leading to heightened anxiety during the reflection phase around one week post-intervention. Additionally, the heightened awareness of health concerns might elicit a temporary emotional response as patients confront new and negative information. The study showed most patients concentrated on treatment^12^ and prognosis^25^ questions, which involved negative consequences and threatening situations, thereby lead to increased anxiety in short-term time. Nevertheless, QPL might reduce anxiety as patients continue to gather more information. Third, the lack of improvement in short-term psychological outcome indicators with a QPL may be attributed to the intervention not being designed to target the psychological component of patients with cancer. To address this unintended effect, incorporating psychological elements into the QPL interventions may be beneficial,^19^ such as providing psychological advice and adding related items of QPL. Additionally, offering healthcare providers training in psychological support and communication prior to consultations, along with including psychological services during consultations and booster interventions, may help alleviate patients' short-term anxiety.^24^^,^^31^ Research suggests that patients' anxiety, nervousness, and fear can affect their ability to use a QPL to ask physicians questions^48^ but incorporating psychological interventions into communication can help alleviate patients’ emotional distress and facilitate communication.^49^ Future research can provide evidence on whether providing psychological intervention content in QPL items and throughout the intervention process can help patients with negative emotions and facilitate patient-physician communication.

Differences in sociodemographic characteristics and QPL interventions can lead to varying effects in the use of QPL. For instance, studies have indicated that older adults tend to have more difficulty engaging in medical decision-making and using QPL effectively.^50^^,^^51^ Moreover, patients at different stages may respond differently to QPL. Patients with advanced cancer, who often have lower spirits and worse physical conditions than early-stage patients, may lack the motivation to read and follow QPL. Similarly, cultural variations across regions also play a role. In Asia, because of the influence of the Confucian culture, many patients may be unwilling to discuss their conditions with healthcare providers.^52^ As a result, these patients often take a more passive role in medical interactions, the effectiveness of QPL in promoting communication and decision-making may be restricted. As for QPL items and contents, patients with cancer may use QPL as a reference to shape the general direction and content of their questions, so different QPL items and contents may influence the effects of the QPL on question asking, consultation length, and patients’ decision-making. Therefore, in oncology clinical practice, it is more advisable to apply QPL with specific items and content tailored to the corresponding characteristics of patients with cancer. This approach is more likely to enhance the effectiveness of QPL.

### Implications for nursing practice and research

The results of our review have implications for clinical oncology practice. First, we recommend to provide QPL with instructions before consultation to help patients ask questions. Second, healthcare providers are often reluctant to adopt QPL due to concerns that they may increase workload,^53^ medical records can prevent physicians from forgetting to use it and ensure more accurate implementation, thereby alleviate healthcare providers' workload. For patients, our study recommends providing QPL at least 10–15 minutes before consulting with healthcare providers to ensure patients have enough time to read the QPL. Additionally, patients' needs vary widely according to diverse cultural backgrounds and personal circumstances,^54^ which may affect the effectiveness of communication between patients and healthcare providers.^48^ An electronic system allows for the delivery of personalized questions tailored to individual patient needs, thereby maximizing the utility of QPLs and enhancing patient engagement in healthcare decisions.^12^ Finally, QPL could be personalized for patients via the web and incorporate multimedia elements such as video or audio recordings to assist patients with dyslexia.

The results of this study have two implications for future research. First, the research population we included was mostly middle-aged and elderly, with intervention sites predominantly located in hospitals for face-to-face consultations. However, findings in previous studies suggested that a QPL's effectiveness in different settings and different patient populations may differ. And studies can conduct interventions in different settings (such as the patient's home or online communication platforms) and to explore the most suitable QPL and standardize its application. Thus, further investigation is warranted to measure the effectiveness of a QPL in the broader and specific scope of patient populations with cancer (such as QPL for pediatric, young adult patients with cancer, and elderly patients with cancer) to prevent increased heterogeneity due to different age groups. Second, the majority of studies reviewed exhibited a high risk of bias due to deviations from established interventions. Although blinding is challenging in clinical settings, these deviations can lead to imbalanced or missing data. Future research should implement stricter blinding protocols, such as using private rooms, to improve outcome accuracy.

### Limitations

However, the study has several limitations. First, there was moderate to large heterogeneity in the population and interventions, and although we used random effects models and subgroup analyses to explore and analyze sources of heterogeneity, caution is still required in interpreting the results. Second, most studies included in our review had a small sample size, which has the potential to reduce the overall power. In addition, language and regional limitations may have led to publication bias, with only three of the 14 studies conducted in Asia and the rest in Europe and the Americas. The use of a QPL was more concentrated in developed countries. Therefore, the results of the review should be interpreted with caution in Asia and in developing countries. In addition, although our review demonstrated the robustness and reliability of the meta-analysis results for most outcomes, the pooled results for some outcomes were affected by differences in QPL intervention facilitators, QPL contents, and measurement tools.

## Conclusions

This study systematically synthesized the characteristics and effectiveness of QPL interventions. Overall, QPL interventions improved patient engagement in shared decision-making, increased the number of questions asked by patients, and enhanced the helpfulness of material to improve patients to ask more questions. Compared with QPL only, QPL with instructions showed superior performance in enhancing asking questions and patients' decision self-efficacy, while QPL only made patients feel more useful. Future research is recommended to refine interventions by identifying key components, addressing implementation barriers in clinical settings, and incorporating psychological support to enhance patient-physician communication and reduce patient anxiety with QPL.

## CRediT authorship contribution statement

**Ruichen Han**: Data Curation, Formal Analysis, Investigation, Visualization, and Writing – Original Draft. **Jiayi Liu**: Formal Analysis, Investigation, Validation, and Writing – Review & Editing. **Jinfeng Ding**: Supervision, Validation, and Writing – Review & Editing. **Jiarui Chen**: Supervision, Validation, and Writing – Review & Editing. **Can Gu**: Supervision, Validation, Writing – Review & Editing. **Ni Gong**: Supervision, Validation, Writing – Review & Editing. **Jinnan Xiao**: Conceptualization, Funding Acquisition, Methodology, Project Administration, Supervision, and Writing – Review & Editing. All participants provided written informed consent.

## Ethics statement

Not required.

## Data availability statement

Data availability is not applicable to this article as no new data were created or analyzed in this study.

## Declaration of generative AI and AI-assisted technologies in the writing process

No AI tools/services were used during the preparation of this work.

## Funding

This work was supported by the Research Project of Hunan Nursing Association (Grant No. HNKYQ202302). The funders had no role in considering the study design or in the collection, analysis, interpretation of data, writing of the report, or decision to submit the article for publication.

## Declaration of competing interest

The authors declare no conflict of interest.
