# Supplementary material for: Effectiveness of question prompt list interventions for patients with cancer: A systematic review and meta-analysis of randomized controlled trials
Source: Asia Pac J Oncol Nurs. 2025 Jul 25;12:100765. doi: 10.1016/j.apjon.2025.100765 (PMC12391282; doi:10.1016/j.apjon.2025.100765)
Supplement: Multimedia component 1 [file mmc1.docx]

**Appendix 1**

Supplementary material table 1: research results for each database 1

Supplementary material table 2: different outcomes from three articles reporting the same study 3

Supplementary material table 3: studies excluded reasons at the final stage 4

Supplementary material table 4: scales or questionnaires 8

Supplementary material table 5: sensitivity analysis 9

**Supplementary material table 1: research results for each database**

| Search nr | Term |
| --- | --- |
| *Concept 1* | |
| #1 | Neoplasms [MeSH] |
| #2 | **Cancer*** OR **Carcinom* OR Malignan* OR Neoplas* OR Tumor* OR Tumour* OR Oncolog* OR lymphoma* OR sarcoma* OR leuk*mia[Title/Abstract]** |
| #3 | #1 OR #2 |
| *Concept 2* | |
| #4 | ‘Question prompt list*’ OR ‘Question prompt sheet*’ OR ‘prompt list*’ OR ‘question prompt*****’ OR ‘prompt sheet*’[Title/Abstract] |
| *Final results* | #3 AND #4 |
| *Final results with filters* | #3 AND #4 *filters:Randomized Controlled Trial , English* |

| **PubMed** | | |
| --- | --- | --- |
| **Search nr** | **Term** | **result** |
| Concept 1 |  |  |
| #1 | Neoplasms [MeSH] | 4,063,153 |
| #2 | "cancer*"[Title/Abstract] OR "carcinom*"[Title/Abstract] OR "malignan*"[Title/Abstract] OR "neoplas*"[Title/Abstract] OR "tumor*"[Title/Abstract] OR "tumour*"[Title/Abstract] OR "oncolog*"[Title/Abstract] OR "lymphoma*"[Title/Abstract] OR "sarcoma*"[Title/Abstract] OR "leuk*mia"[Title/Abstract] | 4,692,679 |
| #3 | #1 OR #2 | 5,545,664 |
| Concept 2 |  |  |
| #4 | "question prompt list*"[Title/Abstract] OR "question prompt sheet*"[Title/Abstract] OR "prompt list*"[Title/Abstract] OR "question prompt*"[Title/Abstract] OR "prompt sheet*"[Title/Abstract] | 370 |
| #5 | #3 AND #4 | 183 |
| Final results with filters | Randomized Controlled Trial, English | 41 |
| **Web of Science** | |  |
| **Type** | **Search Query** | **Results** |
| #1 | TS=(Neoplasms) | 5,982,000 |
| #2 | (TI=(Cancer* OR Carcinom* OR Malignan* OR Neoplas* OR Tumor* OR Tumour* OR Oncolog* OR lymphoma* OR sarcoma* OR leuk*mia)) OR AB=(Cancer* OR Carcinom* OR Malignan* OR Neoplas* OR Tumor* OR Tumour* OR Oncolog* OR lymphoma* OR sarcoma* OR leuk*mia) | 7,086,142 |
| #3 | #1 OR #2 | 8,759,748 |
| #4 | TI=(‘Question prompt list*’ OR ‘Question prompt sheet*’ OR ‘prompt list*’ OR ‘question prompt*’ OR ‘prompt sheet*’) OR AB=(‘Question prompt list*’ OR ‘Question prompt sheet*’ OR ‘prompt list*’ OR ‘question prompt*’ OR ‘prompt sheet*’) | 40,544 |
| #5 | #3 AND #4 | 2,186 |
| #6 | #5 and English (Languages) and Randomized Controlled Trial (Search within topic) | 98 |
| **CINAHL** | |  |
|  | Term | Results |
| #1 | MH Neoplasms | 92,568 |
| #2 | TI (Cancer* OR Carcinom* OR Malignan* OR Neoplas* OR Tumor* OR Tumour* OR Oncolog* OR lymphoma* OR sarcoma* OR leuk*mia) OR AB (Cancer* OR Carcinom* OR Malignan* OR Neoplas* OR Tumor* OR Tumour* OR Oncolog* OR lymphoma* OR sarcoma* OR leuk*mia) | 783,868 |
| #3 | #1 OR #2 | 802,155 |
| #4 | TI (‘Question prompt list*’ OR ‘Question prompt sheet*’ OR ‘prompt list*’ OR ‘question prompt*’ OR ‘prompt sheet*’) OR AB (‘Question prompt list*’ OR ‘Question prompt sheet*’ OR ‘prompt list*’ OR ‘question prompt*’ OR ‘prompt sheet*’) | 794 |
| #5 | #3 AND #4 | 140 |
| #6 | Narrow by Language: - english, Limiters:Randomized Controlled Trial | 18 |
| **Scopus** | |  |
|  | Term |  |
| #1 | TITLE-ABS ( neoplasms ) | 194,272 |
| #2 | TITLE-ABS ( cancer* OR carcinom* OR malignan* OR neoplas* OR tumor* OR tumour* OR oncolog* OR lymphoma* OR sarcoma* OR leuk*mia ) | 5,497,979 |
| #3 | #1 OR #2 | 5,497,979 |
| #4 | TITLE-ABS ( "question prompt list*" ) OR TITLE-ABS ( "question prompt sheet*" ) OR TITLE-ABS ( "prompt list*" ) OR TITLE-ABS ( "question prompt*" ) OR TITLE-ABS ( "prompt sheet*" ) | 872 |
| #5 | #3 AND #4 | 184 |
| #6 | #5 AND ( randomized AND controlled AND trial ) AND ( LIMIT-TO ( LANGUAGE , "English" ) | 144 |
| **PsycARTICLES** | |  |
| #1 | SU Neoplasms | 10,676 |
| #2 | TI (Cancer* OR Carcinom* OR Malignan* OR Neoplas* OR Tumor* OR Tumour* OR Oncolog* OR lymphoma* OR sarcoma* OR leuk*mia) OR AB (Cancer* OR Carcinom* OR Malignan* OR Neoplas* OR Tumor* OR Tumour* OR Oncolog* OR lymphoma* OR sarcoma* OR leuk*mia) | 42,658 |
| #3 | #1 OR #2 | 44,060 |
| #4 | TI (‘Question prompt list*’ OR ‘Question prompt sheet*’ OR ‘prompt list*’ OR ‘question prompt*’ OR ‘prompt sheet*’) OR AB (‘Question prompt list*’ OR ‘Question prompt sheet*’ OR ‘prompt list*’ OR ‘question prompt*’ OR ‘prompt sheet*’) | 263 |
| #5 | #3 AND #4 | 19 |
| #6 | Narrow by Language: - english, Limiters - Methodology: CLINICAL TRIAL | 1 |

**Supplementary material table 2: different outcomes from three articles reporting the same study**

| Study | Outcomes | Measurement tools | Intervention group | | | Control group | | |
| --- | --- | --- | --- | --- | --- | --- | --- | --- |
|  |  |  | Mean | SD | N | Mean | SD | N |
| (Bottacini et al., 2017)* | Satisfaction with the information | Three following questions | 2.985 | 1.35012406 | 158 | 3.285 | 1.422950017 | 150 |
| (Bottacini et al., 2017) | Anxiety | STAI-X1/R | 1.94 | 0.64 | 158 | 1.94 | 0.63 | 150 |
| (Bottacini et al., 2017) | The number of questions asked by patients with cancer | N | 13 | 9 | 158 | 16 | 12.4 | 150 |
| (Buizza et al., 2020)* | Anxiety | STAI-X1/R | 19.52 | 6.3 | 164 | 19.28 | 6.3 | 160 |
| (Buizza et al., 2021)* | The number of questions asked by patients with cancer | N | 13.4 | 9 | 158 | 15.9 | 12.4 | 150 |
|  | The consultation length | N | 47.7 | 19.2 | 158 | 49 | 18.9 | 150 |
| *: indicates the data that was finally included.  STAI-X1/R: a modified version of the STAI-X1 | | | | | | | | |

**Supplementary material table 3: studies excluded reasons at the final stage**

| **NO** | **Author** | **Year** | **Title** | **Excluded reason** |
| --- | --- | --- | --- | --- |
| 1 | M. Friedrichsen | 2007 | Does a prompt list help patients and caregivers to ask questions about cancer prognosis and care?: Commentary | Commentary |
| 2 | R. S. Hebert, R. Schulz, V. C. Copeland and R. M. Arnold | 2009 | Pilot Testing of a Question Prompt Sheet to Encourage Family Caregivers of Cancer Patients and Physicians to Discuss End-of-life Issues | A pilot study |
| 3 | M. Jefford, K. Lotfi-Jam, C. Baravelli, S. Grogan, M. Rogers, M. Krishnasamy, et al. | 2011 | Development and Pilot Testing of a Nurse-Led Posttreatment Support Package for Bowel Cancer Survivors | A pilot study |
| 4 | F. Weijers, C. Veldhoven, C. Verhagen, K. Vissers and Y. Engels | 2018 | Adding a second surprise question triggers general practitioners to increase the thoroughness of palliative care planning: results of a pilot RCT with cage vignettes | A pilot study |
| 5 | A. Walczak, P. N. Butow, J. M. Clayton, M. H. Tattersall, P. M. Davidson, J. Young, et al. | 2014 | Discussing prognosis and end-of-life care in the final year of life: a randomised controlled trial of a nurse-led communication support programme for patients and caregivers | Protocol |
| 6 | L. J. Taylor, P. J. Rathouz, A. Berlin, K. J. Brasel, A. C. Mosenthal, E. Finlayson, et al. | 2017 | Navigating high-risk surgery: protocol for a multisite, stepped wedge, cluster-randomised trial of a question prompt list intervention to empower older adults to ask questions that inform treatment decisions | Protocol |
| 7 | A. Matsuoka, M. Fujimori, B. Narikazu, A. Takashima, T. Okusaka, K. Mori, et al. | 2022 | Geriatric assessment and management with question prompt list using a web-based application for elderly patients with cancer (MAPLE) to communicate ageing-related concerns: J-SUPPORT 2101 study protocol for a multicentre, parallel group, randomised controlled trial | Protocol |
| 8 | Z. Berger, M. Tung, P. Yesantharao, A. Zhou, A. Blackford, T. J. Smith, et al. | 2019 | Feasibility and perception of a question prompt list in outpatient cancer care | Feasibility study |
| 9 | A. Rault, S. Dolbeault, J. Terrasson, C. Bouleuc, P. Cottu, S. Piperno-Neumann, et al. | 2024 | Facilitating patient-oncologist communication in advanced treatment-resistant cancer: development and feasibility testing of a question prompt list | Feasibility study |
| 10 | K. R. Sepucha and J. Belkora | 2007 | Putting shared decision making to work in breast and prostate cancers: Tools for community oncologists | Not RCT |
| 11 | A. Dimoska, M. H. N. Tattersall, P. N. Butow, H. Shepherd and P. Kinnersley | 2008 | Can a "Prompt List" empower cancer patients to ask relevant questions? | Not RCT |
| 12 | A. Dimoska, P. N. Butow, J. Lynch, E. Hovey, M. Agar, P. Beale, et al. | 2012 | Implementing patient question-prompt lists into routine cancer care | Not RCT |
| 13 | J. Mancini, P. N. Butow, C. Julian-Reynier, R. Dring, P. Festy, P. Fenaux, et al. | 2015 | Question prompt list responds to information needs of myelodysplastic syndromes patients and caregivers | Not RCT |
| 14 | T. Kjaer, S. O. Dalton, E. Andersen, R. Karlsen, A. L. Nielsen, M. K. Hansen, et al. | 2016 | A controlled study of use of patient-reported outcomes to improve assessment of late effects after treatment for head-and-neck cancer | Not RCT |
| 15 | J. Arthur, S. Yennu, K. P. Zapata, H. Cantu, J. Wu, D. Liu, et al. | 2017 | Perception of Helpfulness of a Question Prompt Sheet Among Cancer Patients Attending Outpatient Palliative Care | Not RCT |
| 16 | S. van Dulmen, J. A. Driesenaar, J. C. M. van Weert, M. van Osch and J. Noordman | 2017 | PatientVOICE: Development of a preparatory, pre-chemotherapy online communication tool for older patients with cancer | Not RCT |
| 17 | A. Amundsen, S. Bergvik, P. Butow, M. H. N. Tattersall, T. Sørlie and T. Nordøy | 2018 | Supporting doctor-patient communication: Providing a question prompt list and audio recording of the consultation as communication aids to outpatients in a cancer clinic | Not RCT |
| 18 | H. Sungur, N. G. Yılmaz, B. M. C. Chan, M. E. T. C. van den Muijsenbergh, J. C. M. van Weert and B. C. Schouten | 2020 | Development and Evaluation of a Digital Intervention for Fulfilling the Needs of Older Migrant Patients with Cancer: User-Centered Design Approach | Not RCT |
| 19 | H. Okada, T. Okuhara and T. Kiuchi | 2021 | Development and preliminary evaluation of tablet computer-based decision aid for patients participating in cancer clinical trials | Not RCT |
| 20 | J. Al-Mondhiry, S. D'Ambruoso, C. Pietras, T. Strouse, D. Benzeevi, A. C. Arevian, et al. | 2022 | Co-created Mobile Apps for Palliative Care Using Community-Partnered Participatory Research: Development and Usability Study | Not RCT |
| 21 | J. Twigg, A. Kanatas, G. M. Humphris, D. Lowe and S. N. Rogers | 2022 | Risk stratification for poor health-related quality of life following head and neck cancer through the aid of a one-page item prompt list | Not RCT |
| 22 | R. L. Street, Jr., D. J. Tancredi, C. Slee, D. K. Kalauokalani, D. E. Dean, P. Franks, et al. | 2014 | A pathway linking patient participation in cancer consultations to pain control | Not relevant study outcomes |
| 23 | V. C. McLawhorn, J. Vess and B. P. Dumas | 2016 | Integrating a question prompt list on an inpatient oncology unit to increase prognostic awareness? | Not relevant study outcomes |
| 24 | A. Kanatas, D. Lowe and S. N. Rogers | 2022 | Health-related quality of life at 3 months following head and neck cancer treatment is a key predictor of longer-term outcome and of benefit from using the patient concerns inventory | Not relevant study outcomes |
| 25 | M. H. N. Tattersall and P. N. Butow | 2002 | Consultation audio tapes: An underused cancer patient information aid and clinical research tool | Intervention was not QPL |
| 26 | C. Wang, J. Chen, Y. Wang, W. Xu, M. Xie, Y. Wu, et al. | 2021 | Effects of family participatory dignity therapy on the psychological well-being and family function of patients with haematologic malignancies and their family caregivers: A randomised controlled trial | Intervention was not QPL |
| 27 | S. N. Rogers, D. Lowe, V. Highet, G. Dukanovic, C. Lowies, S. Thomas, et al. | 2022 | Patient characteristics and refusal to participate in a head and neck cancer intervention trial: experience of two tertiary UK head and neck cancer centres | Intervention was not QPL |
| 28 | G. A. Taarnhoj, C. Johansen, A. Carus, R. H. Dahlrot, L. H. Dohn, N. H. Hjollund, et al. | 2023 | The iBLAD study: patient-reported outcomes in bladder cancer during oncological treatment: a multicenter national randomized controlled trial | Intervention was not QPL |
| 29 | P. Butow, R. Devine, M. Boyer, S. Pendlebury, M. Jackson and M. H. Tattersall | 2004 | Cancer consultation preparation package: changing patients but not physicians is not enough | Interventions were not only QPL |
| 30 | J. C. van Weert, J. Jansen, P. M. Spreeuwenberg, S. van Dulmen and J. M. Bensing | 2011 | Effects of communication skills training and a Question Prompt Sheet to improve communication with older cancer patients: a randomized controlled trial | Interventions were not only QPL |
| 31 | S. Aranda, M. Jefford, P. Yates, K. Gough, J. Seymour, P. Francis, et al. | 2012 | Impact of a novel nurse-led prechemotherapy education intervention (ChemoEd) on patient distress, symptom burden, and treatment-related information and support needs: results from a randomised, controlled trial | Interventions were not only QPL |
| 32 | R. M. Epstein, P. R. Duberstein, J. J. Fenton, K. Fiscella, M. Hoerger, D. J. Tancredi, et al. | 2017 | Effect of a Patient-Centered Communication Intervention on Oncologist-Patient Communication, Quality of Life, and Health Care Utilization in Advanced Cancer: The VOICE Randomized Clinical Trial | Interventions were not only QPL |
| 33 | R. A. Rodenbach, K. Brandes, K. Fiscella, R. L. Kravitz, P. N. Butow, A. Walczak, et al. | 2017 | Promoting End-of-Life Discussions in Advanced Cancer: Effects of Patient Coaching and Question Prompt Lists | Interventions were not only QPL |
| 34 | A. Walczak, P. N. Butow, M. H. Tattersall, P. M. Davidson, J. Young, R. M. Epstein, et al. | 2017 | Encouraging early discussion of life expectancy and end-of-life care: A randomised controlled trial of a nurse-led communication support program for patients and caregivers | Interventions were not only QPL |
| 35 | S. N. Rogers, C. Allmark, F. Bekiroglu, R. T. Edwards, G. Fabbroni, R. Flavel, et al. | 2021 | Improving quality of life through the routine use of the patient concerns inventory for head and neck cancer patients: main results of a cluster preference randomised controlled trial | Interventions were not only QPL |
| 36 | S. Eggly, N. Senft, S. Kim, E. I. Heath, H. Jang, T. F. Moore, et al. | 2023 | Addressing multilevel barriers to clinical trial participation among Black and White men with prostate cancer through the PACCT study | Interventions were not only QPL |
| 37 | A. Albada, S. Van Dulmen, R. Otten, J. M. Bensing and M. G. E. M. Ausem | 2009 | Development of E-Info geneca: A website providing computer-tailored information and question prompt prior to breast cancer genetic counseling | Inappropriate participant |
| 38 | D. Gyomber, N. Lawrentschuk, P. Wong, F. Parker and D. M. Bolton | 2010 | Improving informed consent for patients undergoing radical prostatectomy using multimedia techniques: a prospective randomized crossover study | Inappropriate participant |
| 39 | S. K. Smith, L. Trevena, J. M. Simpson, A. Barratt, D. Nutbeam and K. J. McCaffery | 2010 | A decision aid to support informed choices about bowel cancer screening among adults with low education: randomised controlled trial | Inappropriate participant |
| 40 | A. Albada, S. van Dulmen, M. G. Ausems and J. M. Bensing | 2012 | A pre-visit website with question prompt sheet for counselees facilitates communication in the first consultation for breast cancer genetic counseling: findings from a randomized controlled trial | Inappropriate participant |
| 41 | A. Albada, S. van Dulmen, P. Spreeuwenberg and M. G. E. M. Ausems | 2015 | Follow-up effects of a tailored pre-counseling website with question prompt in breast cancer genetic counseling | Inappropriate participant |
| 42 | M. L. Schwarze, A. Buffington, J. L. Tucholka, B. Hanlon, P. J. Rathouz, N. Marka, et al. | 2020 | Effectiveness of a Question Prompt List Intervention for Older Patients Considering Major Surgery: A Multisite Randomized Clinical Trial | Inappropriate participant |
| 43 | E. Kalbfell, A. Kata, A. S. Buffington, N. Marka, K. J. Brasel, A. C. Mosenthal, et al. | 2021 | Frequency of Preoperative Advance Care Planning for Older Adults Undergoing High-risk Surgery: A Secondary Analysis of a Randomized Clinical Trial | Inappropriate participant |
| 44 | D. J. Mariano, A. Liu, S. L. Eppler, M. J. Gardner, S. Hu, M. Safran, et al. | 2021 | Does a Question Prompt List Improve Perceived Involvement in Care in Orthopaedic Surgery Compared with the AskShareKnow Questions? A Pragmatic Randomized Controlled Trial | Inappropriate participant |
| 45 | J. L. Ridgeway, S. M. Jenkins, B. J. Borah, V. J. Suman, B. K. Patel, K. Ghosh, et al. | 2022 | Evaluating educational interventions to increase breast density awareness among Latinas: A randomized trial in a Federally Qualified Health Center | Inappropriate participant |
| 46 | A. Schönau, S. Goering, E. Versalovic, N. Montes, T. Brown, I. Dasgupta, et al. | 2022 | Asking questions that matter – Question prompt lists as tools for improving the consent process for neurotechnology clinical trials | Inappropriate participant |
| 47 | M. McDarby, H. I. Silverstein and B. D. Carpenter | 2023 | Effects of a Patient Question Prompt List on Question Asking and Self-Efficacy During Outpatient Palliative Care Appointments | Inappropriate participant |
| 48 | P. N. Butow, S. M. Dunn, M. H. Tattersall and Q. J. Jones | 1994 | Patient participation in the cancer consultation: evaluation of a question prompt sheet | Missing main information |
| 49 | J. M. Clayton, C. Natalia, P. N. Butow, J. M. Simpson, A. M. O'Brien, R. Devine, et al. | 2012 | Physician endorsement alone may not enhance question-asking by advanced cancer patients during consultations about palliative care | Missing main information |
| 50 | I. Henselmans, H. W. M. Laarhoven, P. Maarschalkerweerd, H. C. J. M. Haes, M. G. W. Dijkgraaf, D. W. Sommeijer, et al. | 2020 | Effect of a Skills Training for Oncologists and a Patient Communication Aid on Shared Decision Making About Palliative Systemic Treatment: A Randomized Clinical Trial | Missing main information |
| 51 | S. N. Rogers, C. Allmark, F. Bekiroglu, R. T. Edwards, G. Fabbroni, R. Flavel, et al. | 2020 | Improving quality of life through the routine use of the patient concerns inventory for head and neck cancer patients: baseline results in a cluster preference randomised controlled trial | Duplicate publication |
| 52 | S. N. Rogers, C. Semple, G. M. Humphris, D. Lowe and A. Kanatas | 2020 | Using a patient prompt list to raise concerns in oncology clinics does not necessarily lead to longer consultations | Duplicate publication |
| 53 | C. Dicks, S. N. Rogers, A. Kanatas, D. Lowe, C. McHale and G. Humphris | 2023 | Concerns raised by people treated for head and neck cancer: a secondary analysis of audiotaped consultations in a health services follow-up clinic | Duplicate publication |

**Supplementary material table 4: scales or questionnaires**

| **Domain** | **Scale** | **Purpose** |
| --- | --- | --- |
| **Patient-physician communication quality** | IN-PATSAT32 | Assesses patient’ availability to technology for doctors and nurses, provision of information, patient-physician communication et al. |
|  | mHCCQ | Measures the patient’ s perceptions of their physicians’ autonomy-supportive communication. |
|  | Following questions | Assesses satisfaction of communication with the doctor |
| **Shared Decision-making** | SDM-Q-9 | Measures [Shared Decision Making](https://www.sciencedirect.com/topics/nursing-and-health-professions/shared-decision-making) in clinical encounters. |
|  | PICS | Measures [Shared Decision Making](https://www.sciencedirect.com/topics/nursing-and-health-professions/shared-decision-making) between physicians and patients. |
| **Decision Self-efficacy** | DSES | Measure decision self-efficacy in their ability to make decisions and engage in SDM. |
|  | item of iE-Q | Measure the patients’ self-efficacy in health problems and burdens. |
| **Anxiety** | STAI | Measures patient anxiety. |
|  | STAI-S | Measures the level of trait and state anxiety. |
|  | HADS | Assesses depression and anxiety levels in hospital settings. |
|  | STAI-X1 | Measures patient anxiety. |
|  | STAI-X1/R | Measures patient anxiety. |
| **Satisfaction with the consultation** | PSQ | Assesses Patients’ satisfaction with the consultation. |
|  | Five items | Assesses Patients’ satisfaction with the consultation.. |
|  | 25 items adapted from Roter and Korsch et al. | Measures satisfaction with the consultation. |
|  | PSA | Measures satisfaction with communication. |
|  | IN-PATSAT32 | Measures satisfaction with care. |
|  | A five-item questionnaire | Assesses Patients’ satisfaction with the consultation. |
| **Satisfaction with the information** | The item of iE-Q | Assesses satisfaction with the information. |
|  | Three following questions | Measures satisfaction with the information. |
|  | The item of IN-PATSAT32 | Measures satisfaction with the information. |
| **Helpfulness of the material** | PAQ | Assesses the extent to which patients felt the material helped them. |
|  | three questions | Assesses the usefulness of the material(s). |
|  | Following questions | Assesses helpfulness of the written material(s). |
|  | PrepDM | Measures the efficiency with QPL help patients communicate with physicians. |
|  | Following questions | Assesses easiness to ask questions with QPL. |
|  | The item of iE-Q | Assesses the helpfulness of the material(s). |
| **mHCCQ**: modified Health Care Climate Questionnaire; **SDM-Q-9**: 9-item Shared Decision-Making Questionnaire; **PICS**: Perceived Involvement in Care Scale; **DSES**: Decision Self-efficacy Scale; **SDM**: Shared Decision-making; **HADS**: Hospital Anxiety and Depression Scale; **STAI**: Spielberger State Anxiety Inventory; **STAI-S**: State-Trait Anxiety Inventory-S; **STAI-X1**: State-Trait Anxiety Inventory-X1; **STAI-X1/R**: a modified version of theSTAI-X1; **PSQ**: Patient Satisfaction Questionnaire; **PSA**: Patient Satisfaction Assessment; **IN-PATSAT32**: in-patient satisfaction questionnaire; **iE-Q**: interactional empowerment questionnaire; **PAQ**: Patient Assessment Questionnaire. | | |

**Supplementary material table 5: sensitivity analysis**

| **Variable** | **excluded** | **N, intervention** | **N, control** | **SMD** | **95%CI** | **I^2^** | ***p*-value** |
| --- | --- | --- | --- | --- | --- | --- | --- |
| the number of questions asked | overall | 521 | 500 | 0.24 | 0.00, 0.48 | 69% | 0.05 |
|  | Bruera et al. 2003 | 491 | 470 | 0.24 | -0.02, 0.51 | 73% | 0.07 |
|  | Arthur et al. 2023 | 458 | 433 | 0.28 | 0.01, 0.55 | 72% | 0.04 |
|  | Shirai et al. 2011 | 489 | 469 | 0.27 | 0.01, 0.54 | 72% | 0.04 |
|  | Brown et al.1999 | 501 | 480 | 0.23 | -0.03, 0.48 | 72% | 0.08 |
|  | Bouleuc et al. 2021 | 450 | 429 | 0.23 | -0.04, 0.50 | 71% | 0.10 |
|  | Eggly et al. 2017 | 481 | 460 | 0.22 | -0.04, 0.48 | 71% | 0.10 |
|  | Smets et al. 2012 | 504 | 489 | 0.21 | -0.03, 0.46 | 70% | 0.09 |
|  | Clayton et al. 2007 | 431 | 420 | 0.18 | -0.05, 0.41 | 59% | 0.13 |
|  | Buizza et al. 2021 | 363 | 350 | 0.33 | 0.15, 0.50 | 22% | 0.0002 |
| consultation length | overall | 508 | 567 | 0.19 | -0.01, 0.38 | 57% | 0.07 |
|  | Brown et al. 2001 | 429 | 409 | 0.20 | -0.05, 0.44 | 64% | 0.12 |
|  | Bruera et al. 2003 | 478 | 537 | 0.19 | -0.03, 0.41 | 64% | 0.09 |
|  | Smets et al. 2012 | 491 | 556 | 0.17 | -0.04, 0.37 | 61% | 0.11 |
|  | Bouleuc et al. 2021 | 437 | 496 | 0.14 | -0.07, 0.36 | 55% | 0.18 |
|  | Arthur et al. 2023 | 445 | 500 | 0.24 | 0.03, 0.45 | 55% | 0.03 |
|  | Clayton et al. 2007 | 418 | 487 | 0.12 | -0.07, 0.31 | 43% | 0.21 |
|  | Buizza et al. 2021 | 350 | 417 | 0.25 | 0.05, 0.45 | 41% | 0.01 |
| patient–physician communication | overall | 183 | 187 | 0.11 | -0.09, 0.31 | 0% | 0.29 |
|  | Bouleuc et al. 2021 | 148 | 146 | 0.13 | -0.01, 0.36 | 0% | 0.27 |
|  | Tsai et al. 2022 | 65 | 71 | 0.07 | -0.26, 0.41 | 0% | 0.67 |
|  | Bruera et al. 2003 | 153 | 157 | 0.11 | -0.11, 0.33 | 0% | 0.34 |
| shared decision-making | overall | 140 | 140 | 0.33 | 0.09, 0.56 | 30% | 0.007 |
|  | Tsai et al. 2022 | 22 | 24 | 0.66 | 0.06, 1.25 | Not applicable | 0.03 |
|  | Negarandeh et al. 2023 | 118 | 116 | 0.26 | 0.01, 0.52 | Not applicable | 0.05 |
| decision self-efficacy | overall | 279 | 280 | 0.14 | -0.25, 0.53 | 78% | 0.48 |
|  | Negarandeh et al. 2023 | 257 | 256 | 0.18 | -0.33, 0.70 | 88% | 0.48 |
|  | Zetzl et al. 2020 | 140 | 140 | 0.30 | -0.11, 0.72 | 48% | 0.15 |
|  | Tsai et al. 2022 | 161 | 164 | -0.07 | -0.28, 0.15 | 0% | 0.56 |
| satisfaction with the consultation | overall | 267 | 260 | 0.01 | -0.16, 0.18 | 0% | 0.88 |
|  | Clayton et al. 2007 | 177 | 180 | 0.03 | -0.18, 0.24 | 0% | 0.78 |
|  | Shirai et al. 2011 | 235 | 229 | 0.01 | -0.17, 0.19 | 0% | 0.91 |
|  | Bouleuc et al. 2021 | 232 | 219 | -0.01 | -0.20, 0.17 | 0% | 0.91 |
|  | Arthur et al. 2023 | 204 | 193 | -0.01 | -0.21, 0.19 | 0% | 0.91 |
|  | Bruera et al. 2003 | 237 | 230 | 0.04 | -0.14, 0.22 | 0% | 0.66 |
|  | Smets et al. 2012 | 250 | 249 | 0.02 | -0.15, 0.20 | 0% | 0.81 |
| satisfaction with the information | overall | 332 | 331 | 0.02 | -0.26, 0.29 | 65% | 0.91 |
|  | Bouleuc et al. 2021 | 297 | 290 | -0.05 | -0.37, 0.27 | 74% | 0.74 |
|  | Zetzl et al. 2020 | 193 | 191 | -0.02 | -0.48, 0.45 | 72% | 0.95 |
|  | Bottacini et al. 2017 | 174 | 181 | 0.14 | -0.06, 0.35 | 0% | 0.18 |
| helpfulness of the material | overall | 399 | 395 | 0.48 | 0.16, 0.81 | 77% | 0.004 |
|  | Shirai et al. 2011 | 367 | 364 | 0.46 | 0.08, 0.83 | 81% | 0.02 |
|  | Smets et al. 2012 | 382 | 384 | 0.48 | 0.12, 0.83 | 81% | 0.009 |
|  | Bruera et al. 2003 | 369 | 365 | 0.44 | 0.07, 0.80 | 80% | 0.02 |
|  | Arthur et al. 2023 | 336 | 328 | 0.58 | 0.24, 0.93 | 74% | 0.001 |
|  | Zetzl et al. 2020 | 260 | 255 | 0.56 | 0.19, 0.94 | 72% | 0.03 |
|  | Tsai et al. 2022 | 281 | 279 | 0.35 | 0.08, 0.62 | 50% | 0.01 |
| anxiety  (immediately) | overall | 227 | 227 | 0.09 | -0.10, 0.27 | 0% | 0.35 |
|  | Buizza et al. 2020 | 63 | 67 | 0.22 | -0.13, 0.56 | Not applicable | 0.22 |
|  | Arthur et al. 2023 | 164 | 160 | 0.04 | -0.18, 0.26 | Not applicable | 0.73 |
| anxiety  (about 1 week) | overall | 289 | 354 | 0.17 | 0.02, 0.33 | 0% | 0.03 |
|  | Tsai et al. 2022 | 171 | 238 | 0.15 | -0.05, 0.35 | 40% | 0.15 |
|  | Brown et al. 2001 | 208 | 196 | 0.12 | -0.07, 0.32 | 12% | 0.21 |
|  | Clayton et al. 2007 | 199 | 274 | 0.24 | 0.05, 0.43 | 0% | 0.01 |
| anxiety  (more than 1 week) | overall | 280 | 265 | 0.08 | -0.08, 0.25 | 0% | 0.32 |
|  | Clayton et al. 2007 | 190 | 185 | 0.08 | -0.12, 0.29 | 0% | 0.43 |
|  | Tattersall et al. 2017 | 242 | 239 | 0.12 | -0.06, 0.30 | 0% | 0.19 |
|  | Bouleuc et al. 2021 | 243 | 221 | 0.09 | -0.09, 0.27 | 0% | 0.34 |
|  | Tsai et al. 2022 | 165 | 150 | 0.03 | -0.20, 0.25 | 0% | 0.81 |
